# Further studies are necessary in order to conclude a causal association between the consumption of monosodium L-glutamate (MSG) and the prevalence of metabolic syndrome in the rural Thai population

**DOI:** 10.1186/1743-7075-10-14

**Published:** 2013-01-24

**Authors:** Michael D Rogers

**Affiliations:** 1 International Glutamate Technical Committee, Avenue Jules Bordet 142, B-1140, Brussels, Belgium

**Keywords:** Monosodium glutamate, Intake, Overweight, Metabolic syndrome

## Abstract

Please see related articles and author responses:

http://www.nutritionandmetabolism.com/content/9/1/50

http://www.nutritionandmetabolism.com/content/10/1/10

http://www.nutritionandmetabolism.com/content/10/1/13

**Abstract:**

The article entitled “Monosodium glutamate (MSG) intake is associated with the prevalence of metabolic syndrome in a rural Thai population”, concluded that higher amounts of individual’s MSG consumption are associated with the risk of having the metabolic syndrome and being overweight independent of other major determinants. However, this epidemiological study is the only study indicating such a relationship between MSG intake and the prevalence of metabolic syndrome and there is no direct supporting evidence for a causal relationship between MSG intake and prevalence of metabolic syndrome. This study does not indicate that MSG causes metabolic syndrome. Furthermore, there are several questionable points concerning study methods. Further carefully designed studies taking into account all glutamate sources are necessary to demonstrate the relationship between overweight, metabolic syndrome, MSG intake and umami sensitivity.

## Background

Recently, Insawang *et al*., reported that Monosodium glutamate (MSG) intake is associated with the prevalence of metabolic syndrome in a rural Thai population [1]. However, there is no supporting evidence for a causal relationship between MSG intake and the prevalence of metabolic syndrome. We consider that it is premature to draw any conclusion that MSG consumption increases the risk of metabolic syndrome and overweight. In this commentary, we would like to point out some problems regarding their study and interpretation.

## Discussion

The authors claim that “recent cross-sectional and longitudinal studies in healthy Chinese subjects correlated MSG intake with an increased risk of being overweight irrespective of the total calorie intake and physical activity” and cited two articles from the same research group that suggest an association of MSG intake and overweight in the Chinese population. A problem of these studies is the large difference of MSG intake, one is only 0.33 g/day and the other is 2.2 g/day, which raises questions regarding the accuracy of the MSG intake. Furthermore, they do not mention another study that showed no association between MSG intake and obesity in the Chinese population [2]. In addition, a recent study showing no association between MSG intake and overweight in the Vietnamese population was published [3]. Thus, the results of epidemiological studies on MSG intake and overweight are inconsistent, may be in part because of the difficulties of assessing accurate MSG intake and difference in countries, population and setting. The authors also claim that “animal models support a causative association between obesity and neonatal or maternal administration of high doses of MSG” and cited four animal studies. It has been shown that when neonatal rodents are injected with huge amounts of MSG, blood glutamate levels becomes extremely high, which can develop lesions in certain parts of the brain because the blood–brain barrier in neonatal rodents is immature, and these brain lesions resulted in obesity [4]. It has also been shown that when fasted human subjects ingested MSG in water, consommé or a liquid meal, blood glutamate level were transiently raised [5-7], however, the level is not high enough to develop brain lesions and even this change did not affect the brain because, in humans, blood glutamate does not pass the blood–brain barrier. In addition, the peak blood glutamate levels could be attenuated by other food components [8]. Thus, the circadian variation of blood glutamate level is small during the day in humans ingesting MSG as a food constituent [9]. The fact that the neurotoxicity seen in neonatal rodent studies is not relevant to the safety of MSG used as a food additive for humans has been confirmed by authoratative risk assessment expert bodies of the FAO/WHO, EC and USA [10].

In this study, the authors measured only the additional MSG consumption they provided and did not take into account other sources of glutamate. MSG is only one among many other foods that contains glutamate. There are two forms of glutamate in food, free and protein bound glutamate, protein bound glutamate is digested in the intestine to free amino acids and small peptides, both of which are absorbed into mucosal cells where peptides are hydrolyzed to free amino acids. Therefore, glutamate from added MSG and free glutamate derived from food are chemically identical and are metabolized in the same way in humans ingesting them orally. The human body cannot distinguish glutamate from different sources. Glutamate rich seasonings such as fish and soy sauce, shrimp paste, and MSG containing premix seasonings, are very popular in Thailand and the total amount of glutamate from those seasonings and especially the glutamate from proteins is usually higher than that from MSG. It is very unlikely that exclusively the added glutamate from MSG causes overweight and metabolic syndrome because it is logical to assume that glutamate from other sources also should have the same effect. By and large, it is very unlikely that only one ingredient becomes the cause of obesity and metabolic syndrome, which are complex multifactorial diseases and this epidemiological study does not indicate MSG causes obesity and/or metabolic syndrome. Overall, there is a consensus that obesity is the cumulative result of the net balance of energy intake and expenditure. In this study, there is no association between MSG intake and energy intake or physical activity. Thus, the possible hypothesis that MSG makes food palatable and increases total energy intake is not applied. Several human studies of the elderly have demonstrated that MSG had no effect on total energy intake and body weight, although the effects of MSG on food intake were varied [11-13]. Thus, there isn’t any convincing evidence for a causal relationship between MSG intake and overweight or metabolic syndrome. The BMI median and the median and percentage values of any of the five criteria of ATP III are not significantly different cross-sectionally in association with MSG intake. As a result, these data suggest a different interpretation of the study. Recently, it was reported that obese women have a lower MSG taste sensitivity and prefer higher concentrations than do normal-weight women [14]. Therefore, increased MSG intake may be, in part, a result rather than a cause, and overweight may be a cause rather than a result. There is the possibility that overweight people tend to use more MSG because of their taste preference for higher concentration and this tendency might affect reported inconsistent associations between MSG intake and overweight. In this hypothesis, the association of only MSG among other glutamate sources seems rather convincing. Recently, it has been suggested that glutamate or the taste of glutamate, the umami taste, may play an important role in appropriate food intake [15].

In addition, there are several questionable points regarding the study methods as follows;

1. According to their previous study, subjects who met exclusion criteria were excluded from the 349 subjects resulted in 315 participants [16]. Although the exclusion criteria are the same for both studies, participants were 349 in this study. Participants may be the same because the median MSG intake and interquartile range are the same for both studies, thus, there is a discrepancy about the number of participants.

2. They provided 250 g MSG in a box and measured weight of returned box to assess MSG consumption under the assumption that MSG intake measured during 10 days reflect past MSG intake behavior. This method seems to be similar to the weighted food component measurement which is often used for food consumption studies and thought to be more accurate than other methods such as 24 h food recall or food frequency questionnaire. However, in the usual weighted food component measurement, participants use their own ingredient whereas these participants used free MSG which was given by the investigator in this study. This circumstance is quite different from the usual evaluation for participants, and possibly affected the results. If free MSG is provided, it is very likely that individuals use more MSG than usual because it is free. Thus, this method could not be considered to be in the same category as the usual weighted food component measurement and validation information should be provided.

3. In rural areas of Thailand, multi generations are often living together in the family home. There may be a number of children in each such family home, but the study excluded children under 10 years regardless of the number of children in the calculation, and this possibly affected the MSG consumption data, although the statistical analysis was adjusted for age.

4. None of the median and percentage values of the five criteria of ATP III were individually associated with MSG intake cross-sectionally. Only the percentage of the metabolic syndrome (defined as three or more of the five criteria are met) is associated with MSG intake. This seems to be an oversight. More detailed data should be provided. In addition, the observed associations are very weak with the very small odds ratios for overweight and metabolic syndrome (1.16 and 1.14 respectively) although statistically significant.

5. They chose MSG users only so there is no comparison of the non-MSG users and MSG users.

## Conclusions

This epidemiological study only showed a relationship between MSG intake and the prevalence of metabolic syndrome and there is no direct supporting evidence for a causal relationship. This study does not indicate that MSG causes metabolic syndrome. Further carefully designed studies taking into account all glutamate sources are necessary to demonstrate the relationship between overweight, metabolic syndrome, MSG intake and umami sensitivity.

## Competing interests

M.D.R is the Chairman of the International Glutamate Technical Committee (IGTC), a worldwide research organization having NGO status and carrying out or sponsoring extensive research on the efficacy, application and safety of glutamic acid and its salts especially as used in food. IGTC receives financial support from glutamate producers and users.
